# Are there differences between SIMG surgeons and locally trained surgeons in Australia and New Zealand, as rated by colleagues and themselves?

**DOI:** 10.1186/s12909-022-03560-y

**Published:** 2022-07-02

**Authors:** Ajit Narayanan, Michael Greco, Tina Janamian, Tamieka Fraser, Julian Archer

**Affiliations:** 1 Auckland University of Technology, Auckland, New Zealand; 2 School of Medicine, Griffith University, Brisbane, QLD Australia; 3 CFEP Surveys, Everton Park, QLD Australia; 4 School of Business, University of Queensland, St Lucia, QLD Australia; 5 Education and Innovation, Australian General Practice Accreditation Limited (AGPAL), Brisbane, QLD Australia; 6 Australian General Practice Accreditation Limited (AGPAL), Brisbane, QLD Australia; 7 School of Medicine and Dentistry, Griffith University, Brisbane, Australia

**Keywords:** International medical graduates, Surgery, Colleague feedback, Self-assessment, Professional skills, Network analysis

## Abstract

**Background:**

Representation of specialist international medical graduates (SIMGs) in specific specialties such as surgery can be expected to grow as doctor shortages are predicted in the context of additional care provision for aging populations and limited local supply. Many national medical boards and colleges provide pathways for medical registration and fellowship of SIMGs that may include examinations and short-term training. There is currently very little understanding of how SIMGs are perceived by colleagues and whether their performance is perceived to be comparable to locally trained medical specialists. It is also not known how SIMGs perceive their own capabilities in comparison to local specialists. The aim of this study is to explore the relationships between colleague feedback and self-evaluation in the specialist area of surgery to identify possible methods for enhancing registration and follow-up training within the jurisdiction of Australia and New Zealand.

**Methods:**

Feedback from 1728 colleagues to 96 SIMG surgeons and 406 colleagues to 25 locally trained Fellow surgeons was collected, resulting in 2134 responses to 121 surgeons in total. Additionally, 98 SIMGs and 25 Fellows provided self-evaluation scores (123 in total). Questionnaire and data reliability were calculated before analysis of variance, principal component analysis and network analysis were performed to identify differences between colleague evaluations and self-evaluations by surgeon type.

**Results:**

Colleagues rated SIMGs and Fellows in the ‘very good’ to ‘excellent’ range. Fellows received a small but statistically significant higher average score than SIMGs, especially in areas dealing with medical skills and expertise. However, SIMGs received higher scores where there was motivation to demonstrate working well with colleagues. Colleagues rated SIMGs using one dimension and Fellows using three, which can be identified as clinical management skills, inter-personal communication skills and self-management skills. On self-evaluation, both SIMGs and Fellows gave themselves a significant lower average score than their colleagues, with SIMGs giving themselves a statistically significant higher score than Fellows.

**Conclusions:**

Colleagues rate SIMGs and Fellows highly. The results of this study indicate that SIMGs tend to self-assess more highly, but according to colleagues do not display the same level of differentiation between clinical management, inter-personal and self-management skills. Further research is required to confirm these provisional findings and possible reasons for lack of differentiation if this exists. Depending on the outcome, possible support mechanisms can be explored that may lead to increased comparable performance with locally trained graduates of Australia and New Zealand in these three dimensions.

**Supplementary Information:**

The online version contains supplementary material available at 10.1186/s12909-022-03560-y.

## Background

Given the growing contribution of specialist international medical graduates (SIMGs) in many countries [1–4] and to Australia and New Zealand in particular [5, 6], where it is estimated that between 30 to 40% of the current medical workforce received their medical degree overseas [7], assessing their performance in comparison to their nationally-trained counterparts is important for ensuring consistent attainment of clinical skills [8, 9] as well as interpersonal and communication skills [10, 11]. SIMGs wishing to enter specialist areas may need to achieve certification as specified by specific boards and colleges given differences in exposure to such specialties in different countries [12, 13]. In Australia and New Zealand, for instance, the Royal Australasian College of Surgeons (RACS) is accredited by the Australian Medical Council (AMC) and the Medical Council of New Zealand (MCNZ) to train surgeons and maintain surgical standards. RACS oversees an assessment process for specialist recognition of SIMGs which evaluates the training, qualifications, and experience of SIMGs for comparability with an Australian or New Zealand trained surgeon. RACS may grant an SIMG a pathway to Fellowship based on the SIMG’s formal postgraduate specialist qualifications in surgery and relevant experience.

There has been previous research on whether patients perceive differences between IMGs and locally trained doctors in the context of Australian general practice, with no statistical difference in quality of experience but a trend indicating that Australian-trained doctors cared more and provided greater understanding [14]. There has also been research on support mechanisms, including colleague and training support, for helping to prepare IMGs for general clinical practice in their new countries [15]. But there is very little understanding of how colleagues see differences, if any, between locally trained specialists and their SIMG counterparts in areas such as surgery. If there are differences, these could usefully be addressed as part of specialist certification processes and follow-up professional development programmes to ensure consistency of performance within that specialism.

Another important aspect of performance assessment is self-assessment to promote reflection on personal performance [16]. Self-assessment supports the ability to assess one’s own work critically, with research showing that physicians may require professional development to accurately self-assess [17]. While there is research indicating that weaker doctors tend to overrate themselves [18], no relationship between self-assessment and formal assessment of clinical competence was found among junior Australian doctors [19]. However, with the specialist area of surgery, there has been no previous research on identifying whether there is any difference in the self-assessments of SIMG and locally trained surgeons, and how such self-assessment is related to colleague feedback. Any differences in colleague scores and self-scores may identify areas for enhancing professional growth and performance in that specialist area.

There are two aims to the study reported below: (a) to gather and analyse colleague feedback on SIMG surgeons and locally trained surgeon Fellows to identify any perceived differences in clinical, interpersonal and communication skills, and (b) to evaluate whether there are any differences in self-evaluations by SIMG surgeons and surgeon Fellows that may help to explain perceived colleague differences as well as identify areas for enhanced continuing professional development.

## Methods

### Questionnaire

The method for obtaining feedback from colleagues was through a modified version of the Colleague Feedback Evaluation Tool (CFET) designed and developed by CFEP Surveys, and previously validated by the General Medical Council for possible use as part of the revalidation of GPs in the UK [20]. The original CFET tool was modified (CFET-RACS) to reflect more closely the medical and technical expertise, judgement, and inter-personal skills expected of a surgeon. In particular, the CFET-RACS was aligned to the nine surgical competencies before they were recently updated to ten surgical competencies [21]. CFET-RACS consists of 27 items with Likert scale values 1–5 (1 = ‘Poor’, 2 = ‘Fair’, 3 = ‘Good’, 4 = ‘Very good’, 5 = ‘Excellent’, and 6 = “Unable to comment”). Two additional questions asked for comments on other strengths of the surgeon and how the surgeon could become more effective. Qualitative analysis of colleague comments can help identify areas where targeted interventions are likely to be beneficial [22], and some general findings and conclusions are included here. Colleagues were also asked to state what sort of colleague they were: RACS Fellow, trainee or SIMG under assessment (Rater Type RT1); other registered health practitioner (RT2); or other professional colleague (RT3). The list of Abbreviations includes a description of each of the 27 items and their questionnaire (Q) identifier used when reporting results.

The participating surgeons were also asked to complete the CFET-RACS for self-evaluation (SE) purposes as well as make comments about their own strengths and possible areas for improvement.

### Data collection

Participating surgeons were advised to nominate at least 15 raters from a range of colleagues with whom they work, including medical and non-medical peers (other clinicians and administrative staff). Nominated colleagues were then emailed the questionnaire for completion, with two follow-up reminders, if required. The colleague questionnaire was completed online via a secure web portal. The questionnaires were processed by CFEP Surveys in Brisbane, Australia. Online validation and verification were conducted before being downloaded to in-house software systems. The dataset was exported as a Microsoft Excel Spreadsheet to an SPSS database (SPSS for Windows Version 25) and cleaned and checked prior to data analysis. After final data collection, participating surgeons received feedback reports preserving rater anonymity and confidentiality for the purpose of supporting their reflections on performance. Their reports included both quantitative tables and graphs with benchmarks (broad indications of how they were rated in comparison to the cohort as a whole), as well as qualitative comments provided by raters.

### Statistical analysis

To aid analysis and interpretation the intervals between each scale point were assumed to be equal and the points equate to percentages (‘poor’ = 20%, ‘fair’ = 40%, ‘good’ = 60%, ‘very good’ = 80%, ‘excellent’ = 100%). The exploratory analysis was at two levels for colleague data. At the raw item score provided by colleagues; and at the aggregated, surgeon level (item mean scores received from colleagues). The sampling method resulted in the data being unbalanced because the number of raters per ratee was variable, fully nested because raters had unique ratees, and uncrossed because raters provided only a single rating.

Cronbach’s α is reported below as a measure of questionnaire reliability, but the results should be interpreted cautiously because of the unbalanced, fully nested and uncrossed sampling method. Cases with missing values are also excluded from Cronbach’s α, which can lead to significant loss of information [23]. A signal-to-noise ratio (SNR) formula for data reliability is used [24] which combines raw and aggregated item, rater and subject variances, with consideration to the average number of raters per ratee, to address the issues introduced by the sampling strategy. The SNR formula includes all cases. Single measures intraclass coefficients (ICCs) are used to check for inter-rater reliability for this specific study. ICCs of 0.4–0.6, 0.6–0.8, 0.8 + considered to show moderate, good and very good agreement, respectively [25].

Statistical analysis included analysis of variance (ANOVA) to test for differences in item ratings and averages in and between SIMG and Fellow data, and regression to control for the effects of demographic factors on colleague raw scores. Principal component analysis (PCA) is used to identify how much of the total variance in the aggregated (surgeon) level can be explained by linear combinations of items forming uncorrelated components. Exploratory PCA can check whether patterns of responses to the two surgeon types share similar components or dimensions.

Psychometric network analysis [26] is used to explore relationships between variables, where nodes represent items and edges the associations between items. Partial correlations are used for edge calculation and regularized to remove spurious connections through least absolute shrinkage and selection operator (LASSO) by minimizing the extended Bayesian information criterion (EBIC) [27]. Network analysis provides three centrality measures (closeness, betweenness, degree) for determining importance of nodes [28]. Additional file 1: Statistical Methods contains more information on the statistical methods used in this study. All statistical analysis was performed with SPSS v27 and SPSS v28, and network analysis through JASP v0.14.1.

## Results

### Colleague feedback (raw score)

There were 1728 colleague responses to 96 SIMGs and 406 colleague responses to 25 Fellows, resulting in 2134 colleague responses to 121 surgeons in total. The average colleague item score was a high 92.35% across all 27 items (91.94 lower CI, 92.78 upper CI, median 96.59, 25^th^ percentile 88.0, 75^th^ percentile 100). 689 colleagues (32.3%) provided a rating of 100%. The proportions of colleague rater type were similar for both SIMGs and Fellows (26.9% and 28.6% RT1; 47.2% and 46.6% RT2; and 19.3% and 20.9% RT3; 6.7% and 3.9% not declaring their colleague status). There was no statistically significant difference in item scores provided by Rater type (RT1 average = 91.84%; RT2 average = 92.42%; RT3 average = 92.98%; *p* = 0.21). Rater type contributed just 0.7% of the variance in average colleague score (adjusted R^2^ = 0.007) using controlled regression, with the 27 items contributing the remaining 99.3%.

The average missing value rate was 9.3% (SIMG colleagues = 9.6%, Fellow colleagues = 8.3%), with the highest rates for Q8 (23.9%, SIMG colleagues = 24.6%, Fellow colleagues = 20.9%), Q27 (19.6%, 20.2%, 17%), Q15 (16.9%, 17.7%, 13.8%), Q25 (15.4%, 17.7%, 14.3%), Q26 (15.8%, 15.5%, 17.2%) and Q21 (13.0%, 13.3%, 16.5%). As a result of these missing values, there were 936 (54.2%) and 218 (53.7%) fully completed colleague questionnaires for SIMGs and Fellows, respectively.

For SIMG colleague scores, Cronbach’s alpha was 0.98 (*n* = 936) and for Fellow colleagues 0.96 (*n* = 218), with overall average alpha of 0.98. The high internal questionnaire consistency was confirmed by the lack of improvement in alpha if any item was removed. One-way random ICC was moderately strong overall (0.62, 95% confidence intervals of 0.60 and 0.64) and higher for SIMG colleagues (0.65, 0.63, 0.673) than for Fellow colleagues (0.49, 0.44, 0.54), indicating better agreement among SIMG colleagues on how the items were to be interpreted. Average inter-item correlations were 0.65 for IMG colleagues and 0.49 for Fellow colleagues. Data reliability as calculated by SNR was 0.86, indicating that 86% of the variance in the data was likely to be true variance based on colleague scores with the remainder due to noise.

Missing value analysis showed that missing values were not missing completely at random (Little’s test ≤ 0.001). Imputation was performed using linear regression resulting in 2134 fully completed colleague questionnaires with an average colleague score of 92.20% (in comparison to 92.35% without imputation; 91.73 lower CI, 92.62 upper CI, median 96.23, 25^th^ percentile 87.68. 75^th^ percentile 99.69. After imputation the number of colleagues providing overall scores of 100% reduced to 462 (21.6%). Cronbach’s alpha remained 0.98 for SIMG colleague scores and rose slightly to 0.97 for Fellow colleague scores from 0.96. ICC rose slightly to 0.65 for IMG colleagues scores and 0.50 for Fellow colleague scores (previously 0.62 and 0.49, respectively). Average inter-item correlation remained at 0.65 for IMG colleagues and rose slightly to 0.50 from 0.49 for Fellow colleagues. The full data set including imputed values was used wherever possible to ensure no loss of any colleague ratings.

### Colleague feedback (aggregated)

The average surgeon score was 92.45 (91.45 lower CI, 93.35 upper CI, median 93.53, 25^th^ percentile 90.35, 75^th^ percentile 96.04). The average number of raters per surgeon was 17.64 (minimum 9, maximum 38, median 15). SIMGs received a slightly lower average score from colleagues (92.34%) in comparison to Fellows (92.87%, Additional file 2: Supplementary Table 1). Item by item these differences were significant (p ≤ 0.05). The largest differences in favour of Fellows were in Q1 (+ 2.36%) and Q7 (+ 2.26%). SIMGs received higher scores in Q10 (+ 1.97%), Q23 (+ 1.56%) and Q24 (+ 1.14%). Also, SIMGs received significantly lower minimum scores on all items (average 70.43%) than Fellows (81.12%, *p* < 0.001). Maximum scores received from colleagues were nearly all 100% for both types of surgeon.

The Kaiser–Meyer–Olkin (KMO) test showed 0.97 and Bartlett’s test of sphericity less than 0.001, indicating suitability for principal component analysis. After splitting by surgeon type, a single component was found for SIMGs accounting for 88% of the variances. For Fellows, three components were found accounting for 84% of the variance (Additional file 2: Supplementary Table 2). For Fellows, the first component (42% of variance explained) covered items Q10 to Q24 and Q26; the second (30%) Q1 to Q9, Q25 and Q27; and the third (12%) was a single item component (Q12). On the basis of these loadings for Fellows, component 1 can be labelled *Inter-personal skills*, component 2 *Clinical management skills*, and component 3 *Self-management skills*.

**Fig. 1:**
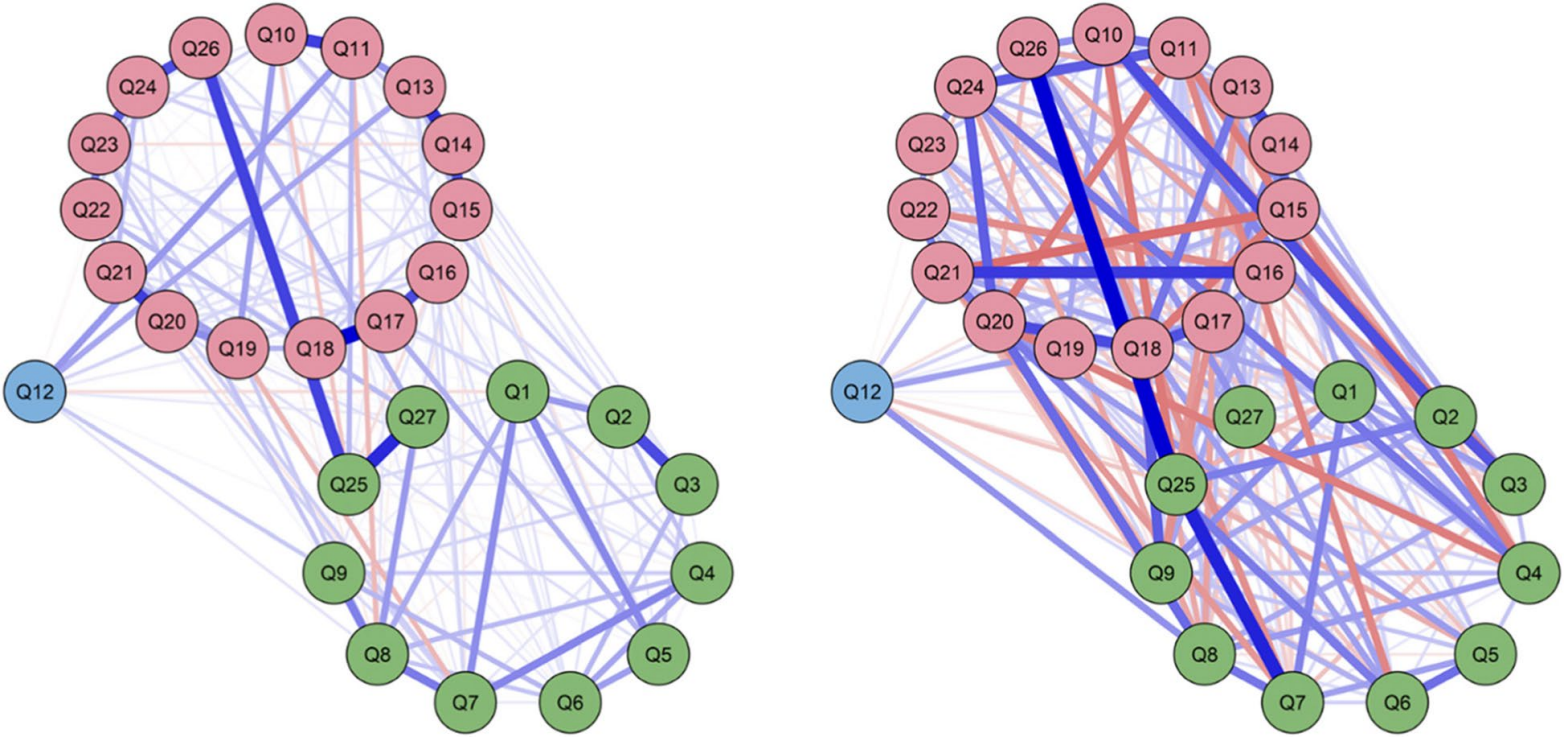
Network visualisations of regularized partial correlations from colleague ratings for SIMGs (left) and Fellows (right). The associations for the one SIMG component are visualised using the three components found for Fellows at the aggregated level (green for Clinical management, pink for Inter-personal skills, and lilac for Self-management). See Table 1 and Abbreviations for meanings of Q1-Q27. Line thickness reflects strength of association, with blue positive and red negative

**Fig. 2:**
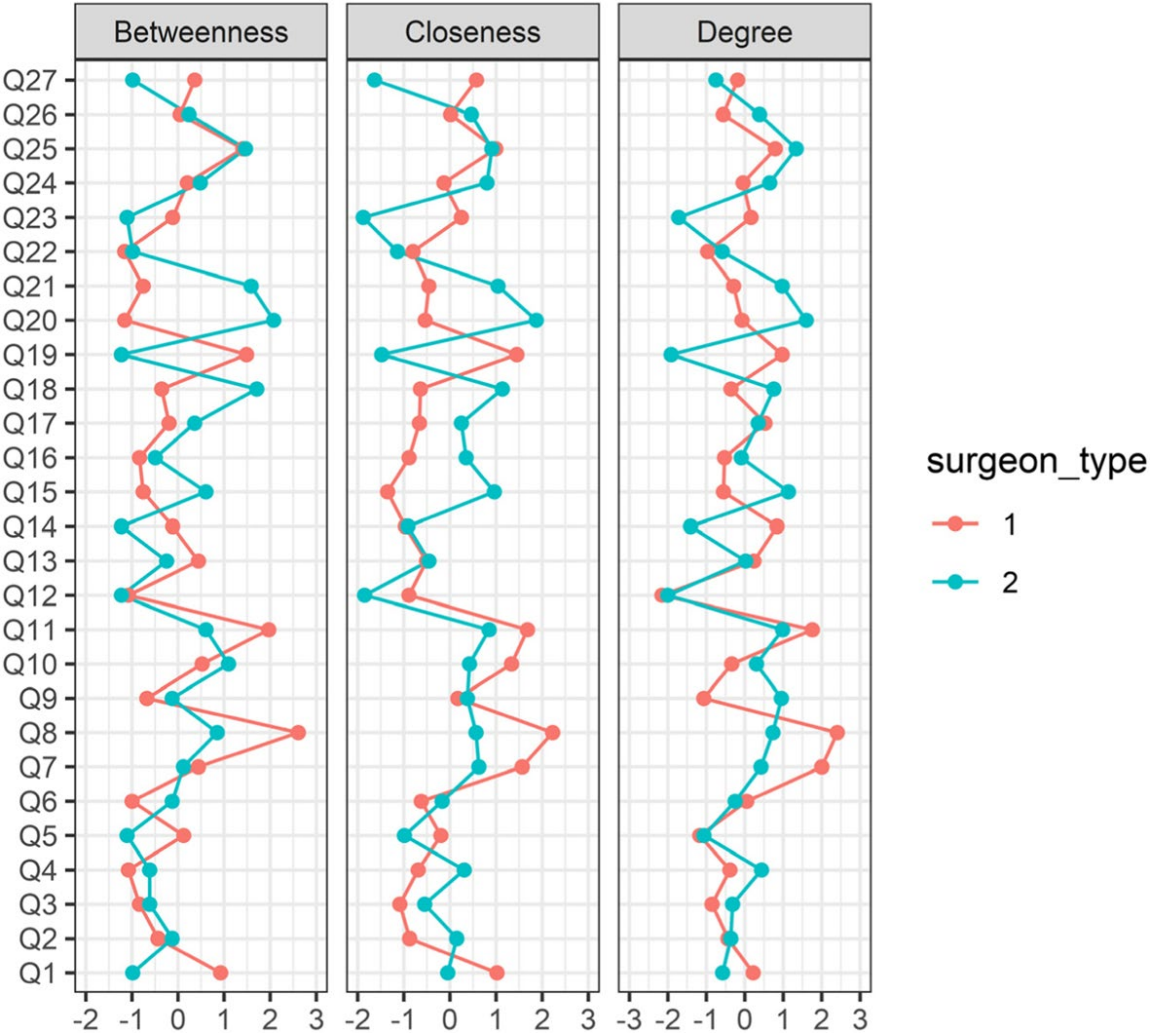
Centrality plots of Betweenness, Closeness and Degree for colleague-rated networks in Fig. 1, with surgeon_type 1 = SIMGs and surgeon_type 2 = Fellows

The average inter-item correlation for SIMGs was 0.871 and 0.648 for Fellows. Network visualisations by surgeon type can be found in Fig. 1. Centrality plots for these networks are in Fig. 2 for item-by-item comparison. Table 1 provides summed centrality scores for each item.

**Table 1:** Summed network centrality scores of Betweenness, Closeness and Degree for SIMGs and Fellows as rated by Colleagues (Fig. 2) and Self (Fig. 4)

Summed Centrality				
	**Colleagues**		**Self**	
***Item***	**SIMGs**	**Fellows**	**SIMGs**	**Fellows**
Q1 Demonstrating medical skills and expertise	2.16	-1.61	0.41	-3.48
Q2 Monitoring and evaluating care	-1.75	-0.34	1.74	-2.12
Q3 Managing safety and risk	-2.77	-1.48	-1.72	3.83
Q4 Considering options	-2.15	0.13	-0.04	0.77
Q5 Planning ahead	-1.24	-3.15	-0.98	1.45
Q6 Implementing and reviewing decisions	-1.57	-0.54	1.27	0.28
Q7 Recognising where surgery may be needed	4.02	1.17	2.88	-4.14
Q8 Maintaining dexterity and technical skills	7.24	2.15	0.84	-0.89
Q9 Defining scope of practice	-1.57	1.21	-0.83	-1.60
Q10 Having awareness and insight	1.52	1.83	-2.90	6.74
Q11 Observing ethics and probity	5.41	2.44	-1.54	0.07
Q12 Maintaining health and well-being	-4.12	-5.08	-4.36	-0.02
Q13 Caring with compassion and respect for patient	0.18	-0.68	2.50	1.26
Q14 Meeting patient, carer and family needs	-0.25	-3.55	-0.06	-1.57
Q15 Responding to cultural and community needs	-2.66	2.71	-3.96	2.55
Q16 Gathering and understanding information	-2.25	-0.23	-4.41	0.53
Q17 Discussing and communicating options	-0.33	0.96	2.72	-3.18
Q18 Communicating effectively	-1.35	3.60	4.53	0.83
Q19 Documenting and exchanging information	3.91	-4.62	-1.36	3.80
Q20 Establishing a shared understanding	-1.77	5.55	1.26	-1.48
Q21 Playing an active role in clinical teams	-1.51	3.60	0.35	-2.35
Q22 Setting and maintaining standards	-2.92	-2.71	2.64	-3.40
Q23 Leading that inspires others	0.30	-4.70	1.97	-2.99
Q24 Supporting others	0.03	1.94	0.49	-1.72
Q25 Showing commitment to lifelong learning	3.20	3.71	-2.36	-0.54
Q26 Teaching, supervision and assessment	-0.50	1.09	-5.00	3.54
Q27 Improving surgical practice	0.76	-3.37	5.93	3.81

Of the 406 colleague responses to Fellows and 1728 colleague response to SIMGs, a total of 258 (63.5%) and 1181 (68.3%) included comments on surgeons’ strengths respectively. For both cohorts, the most common colleague response regarded personal attributes/behaviour, with professional, approachable, compassionate, caring and kind being the top five qualities [22]. There were considerably fewer responses of colleagues when asked to comment on how Fellows and SIMGs could be more effective with 142 (35.0%) and 658 (38.1%) statements, respectively. Of the 142 responses received by colleagues recommending improvements for Fellows’ effectiveness, 33.8% had no suggestions or indicated continuation of current conduct. A higher proportion (43.5%) of colleagues had no suggestions or to continue as normal for SIMGs. Key themes that were identified included suggestions on personal attributes, behavioural changes, medical and technical expertise, time management, and communication [22].

### Self-evaluation

Ninety-eight SIMGs and 25 Fellows provided self-evaluation scores (123 in total). The overall item self-evaluation score was 77.83% (lower CI 75.69, upper CI 79.99, median 79.26) in comparison to the colleague average item score of 93%, with SIMGs giving themselves an average and statistically significant (*p* < 0.001) 3.7% higher self-evaluation scores than Fellows (78.58 and 74.84, respectively, Additional file 2: Supplementary Table 3). The biggest differences were in Q24 (+ 9.5%), Q23 (+ 7.31%), Q26 (+ 6.6%) and Q27 (+ 6.5%). The correlation between the average colleague score and the average self-score was a non-significant 0.011 (*p* = 0.112).

**Fig. 3:**
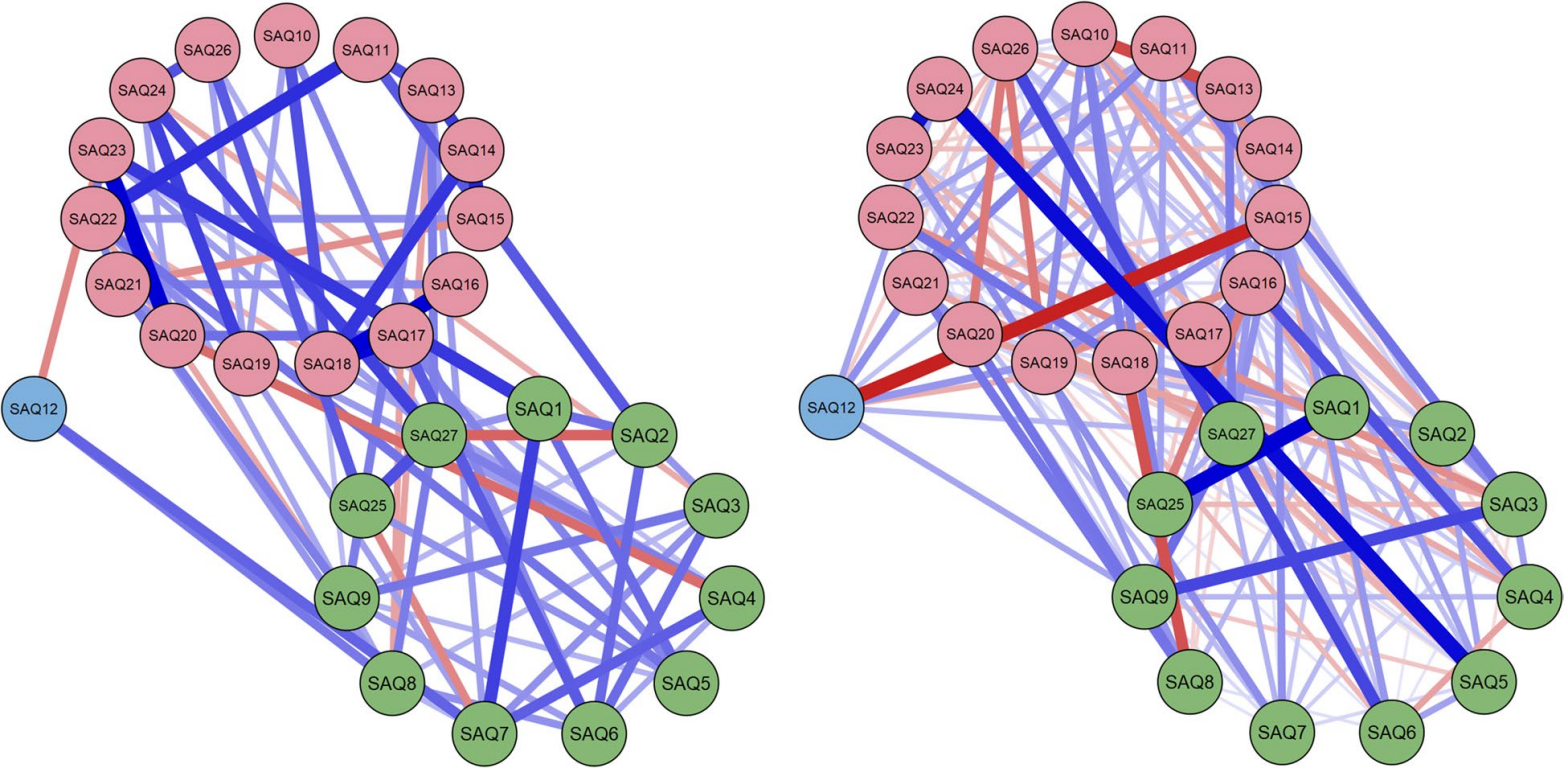
Network visualisations of self-assessment regularized partial correlations, with SIMGs on the left and Fellows on the right (green for Clinical management, pink for Inter-personal skills, and lilac for Self-management). See Table 1 and Abbreviations for meanings of Q1-Q27 in the context of self-assessment (SA). Line thickness reflects strength of association

**Fig. 4:**
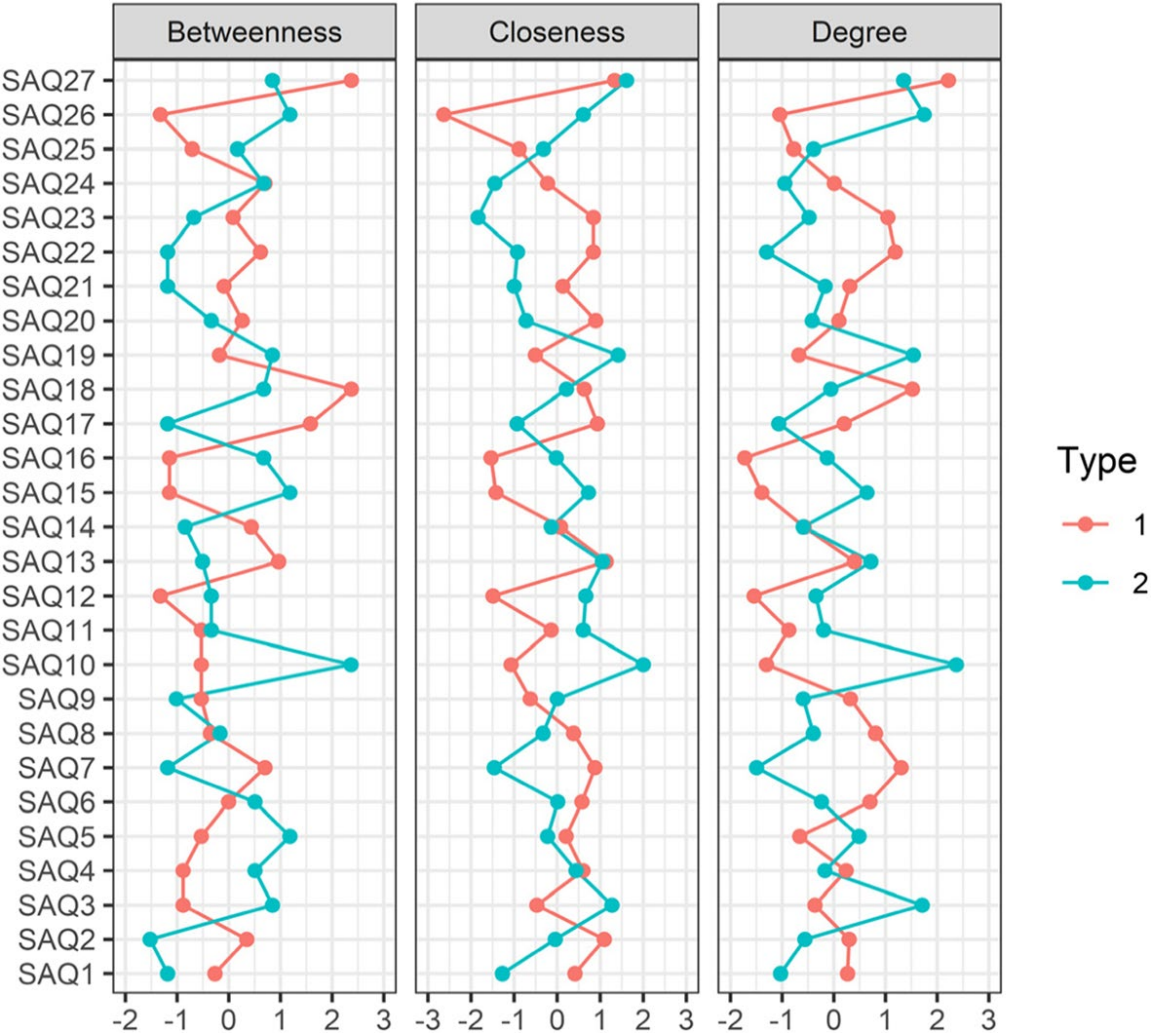
Centrality plots of Betweenness, Closeness and Degree for self-evaluation networks in Fig. 3, with surgeon_type 1 = SIMGs and surgeon_type 2 = Fellows

Two Fellows gave themselves the lowest average of 29% and 39%, and a third 56%. Seven SIMGs averaged the lowest 60%. Two surgeons (one SIMG, the other Fellow) scored themselves at 100% and two at 99% (both SIMG). Additional file 2: Supplementary Fig. 1 compares self-evaluation item scores with colleague scores by surgeon type. The average inter-item correlation was 0.61 for SIMGs and 0.70 for Fellows. Self-assessment networks for the two surgeon groups are shown in Fig. 3, with their centrality plots in Fig. 4 and summed centrality in Table 1.

Self-evaluation on how to become more effective resulted in 77 SIMGs (78.6%) and 13 Fellows (52%) provided responses to the open-ended question. Medical/technical expertise and personal attributes were among the top three themes for both Fellows and SIMGs. Additional key themes included time management SIMGs and teamwork/collaboration for Fellows. Regarding self-evaluation of strengths, of the 98 SIMGs and 25 Fellows who provided self-evaluations, 73 (74.5%) and 14 (56%) respectively completed the open-ended questions regarding their strengths. Similar key themes were identified by both cohorts with team involvement, personal attributes and medical/technical expertise being the top three themes [22].

## Discussion

Colleagues rated SIMGs and Fellow surgeons highly (average item score 92%, which is just over half-way in the ‘very good’ to ‘excellent’ range). No statistically significant difference was found in scores by rater type, which contributed less than 1% of the variance to average colleague score. The high questionnaire consistency (average α = 0.97) and data reliability (SNR = 0.86) of raw-score colleague responses provide some assurance for the reliability of analysis at the surgeon level after aggregation.

Interestingly, colleagues were more inclined to provide comments on SIMGs and Fellows’ strengths than to suggest how they could improve. A large proportion of the improvement suggestions focused on continuation of current conduct rather than recommendations of qualities to improve. A greater percentage of SIMGs completed the additional open-ended questions on their strengths and areas for improving effectiveness in contrast to Fellows (74.5% vs 56.0% and 78.6% vs 52.0% respectively). This could be a reflection of the greater number of SIMGs that initially completed the self-evaluation questionnaire in contrast to Fellows. Qualitative analysis found that medical/clinical expertise, personal attributes/behaviour, team work/collaboration and communication were key themes identified as both strengths and areas for improvement for both SIMGs and Fellows.

The relatively high proportion of missing values for Q8 (Maintaining dexterity and technical skills, 24%), Q27 (Improving surgical practice, 20%), Q15 (Responding to cultural and community needs, 17%), Q25 (Showing commitment to lifelong learning), Q26 (Teaching, supervision and assessment, 15%) and Q21 (Playing an active role in clinical teams, 14%) indicates that many colleagues were not able to observe all aspects of a surgeon’s performance before scoring. These items may need to be reviewed before inclusion in subsequent studies to improve the proportion of fully completed questionnaires (54% in this study).

After aggregation, a small but statistically significant difference of 0.53% (Additional file 2: Supplementary Table 1) was found in the item scores received by the two types of surgeon. Fellows received higher scores of 2.4% in Q1 (Demonstrating medical skills and expertise) and 2.3% in Q7 (Recognizing where surgery is needed). These differences may be due to greater emphasis on these aspects during local medical training. In contrast, SIMGs received 2% higher scores in Q10 (Having awareness and insight), 1.6% in Q23 (Leading that inspires others) and 1.1% in Q24 (Supporting others). These differences may be due to greater motivation and desire to demonstrate working well with colleagues. The main reason for the overall difference in scores was that SIMGs performed worse than Fellows at the very bottom end (83.96% and 89.46% for bottom 10^th^ percentiles, respectively).

If combined and averaged colleagues scores are interpreted as representing a collective ‘objective’ measure of performance of individual performance, the lack of correlation or very weak correlation between self-assessment scores and objective scores (non- significant 0.11) is not surprising [29]. However, the underestimate of self-performance by an average of 14% against objective performance is noteworthy, given previous research indicating that self-assessment can lead to unrealistic optimism, especially in the workplace where overconfidence and overrating tend to dominate [29, 30]. SIMGs gave themselves just under 4% higher scores then Fellows, but overall their scores were still well below their colleagues’ ratings. There could be several reasons for this self under-assessment. First, previous findings were reported at a relatively early stage of self-assessment protocols being introduced into medical professional performance development and, with time, medical professionals have learned to moderate their self-assessments in the light of regular feedback and growing ability to self-reflect in the context of such feedback. Second, surgery training and assessment can involve a high degree of observation in accredited training posts under monitor supervision. Surgeons may be aware of the need to identify limitations and competence boundaries as part of this training and this may lead to an over-emphasis on areas for improvement. Third, the questionnaire used for self-assessment in this study is the same as those used by colleagues, and there may be a heightened sense of “seeing themselves as others see them” which may lead to more critical self-judgements. Previous research using the same questionnaire for both self and colleague assessments with general practitioners also reported similar self under-assessment of nearly 12% [31]. Also, surgeons submitted a list of colleagues to provide ratings. An awareness of who might be providing ratings may help to moderate responses in a way that totally anonymous and unknown raters would not. This awareness may lead to a form of “modesty bias” [32] peculiar to medical performance peer review involving chosen colleagues. Finally, it is possible that colleagues provided over-inflated ratings and surgeons’ self-ratings are more accurate. However, the average of nearly 18 raters per surgeon would make this unlikely.

PCA identified one component for SIMGs and three for Fellows (Additional file 2: Supplementary Table 2). For Fellows, the three components corresponded to Inter-personal skills, Clinical management skills and Self-management skills. These three components for Fellows are consistent with those found in other peer-based studies in medical education [31, 33]. The average inter-item correlation for SIMGs was higher (0.87) in comparison to Fellows (0.65), indicating greater homogeneity and single-dimensionality of ratings for SIMGs. The single dimension for SIMGs could be due to a number of factors, including previous training programmes in other countries not providing opportunities through more focused feedback to enhance competencies involving these three dimensions. The networks of Fig. 1 indicate stronger within-dimension and between-dimension links for Fellows than for SIMGs, providing some evidence that Fellows may have had more opportunity to develop these three sets of skills in parallel. Also, centrality measures (Table 1) show that Fellows and SIMGs differ most in items 15, 18, 19 and 20 (Cultural and community needs, Communicating effectively, Documenting and exchanging information, Establishing a shared understanding). These differences could reflect settling into new environments and developing working relationships that may differ in style and culture from previous environments. Follow-up studies may show whether these differences are temporary and to be expected of SIMGs as part of their re-establishment process.

Network analysis (Fig. 1) revealed similarities and differences across centrality profiles as rated by colleagues. For SIMGs, the highest item on all three measures (summed centrality score of 7.24, Table 1) was Q8 (Maintaining dexterity and technical skills), followed by Q7 (4.02, Recognizing where surgery may be needed) and Q11 (5.41, Observing ethics and probity). For Fellows, Q20 (5.55, Establishing a shared understanding) was strongest, followed by Q25 (3.71, Showing commitment to lifelong learning), Q18 (3.60, Communicating effectively) and Q21 (3.60, Playing an active role in clinical teams). The least central for both groups was Q12 (Maintaining health and well-being, − 4.12, − 5.08).

Both SIMGs and Fellows gave themselves an average 14% lower score than their colleagues, with SIMGs giving themselves a statistically significant 3.7% higher score than Fellows (Additional file 2: Supplementary Table 3). SIMGs rated themselves better than their Fellow counterparts in particular on Q24 (Supporting others, + 10%), Q23 (Leading and inspiring others, + 7%), Q26 (Teaching, supervision and assessment, + 7%) and Q27 (Improving surgical practice, + 7%, Additional file 2: Supplementary Fig. 1). These higher ratings could be due to greater prior experience in these areas (perhaps 50% of IMGs generally have had between 6 to 15 years post-residency experience [34]) as well as desire to demonstrate willingness to contribute as team members. Enhanced professional development may provide SIMGs with opportunities to gain further experience with self-assessment.

When looking at the networks of SIMG and Fellow self-assessment (Fig. 4) and summed centrality (Table 1), the most negative scores could be interpreted as areas where surgeons feel they may need more support in terms of personal development for improved connectedness of professional aspects. So, for instance, SIMGs may benefit from enhanced personal development support for SAQ26 (Teaching, supervision and assessment, − 5.00), SAQ16 (Gathering and understanding information, − 4.41), SAQ12 (Maintaining health and well-being, − 4.36) and SAQ15 (Responding to cultural and community needs, − 3.96). Similarly, Fellows may benefit from enhanced support in SAQ7 (Recognizing where surgery may be needed, − 4.14), SAQ1 (Demonstrating medical skills and expertise, − 3.48) and SAQ22 (Setting and maintaining standards, − 3.40).

Finally, the high proportions of 100% ratings at the raw score level (32% before imputation, 22% after imputation) can produce major ceiling effects for analysis conducted at that level, including difficulty in identifying accurate measures of central tendency and distinguishing groups. These ceiling effects were moderated at the aggregated level with an average of nearly 18 colleague ratings per surgeon, leading to small but significant differences being found. Future applications of the questionnaire will need to address the tendency for colleagues to provide maximum ratings as well as encourage more comment of a constructive but critical nature. It is also possible that colleague responses will show more variation with repeated exposure to the instrument and appreciation that subjects may benefit from feedback that helps to improve their performance. Given that this is the first time such an instrument has been designed and developed for use in the surgical domain to identify possible differences between Fellows and SIMGs, there is still room for development and improvement for the benefit of all surgeons.

In conclusion, a clear difference has been found in performance dimensions underlying ratings for SIMGs and Fellows, with results of this study indicating that colleagues do not differentiate between the clinical management, inter-personal and self-management skills of SIMGs in the same way as they do for Fellows. Investigating the reasons for this lack of differentiation will be important for identifying the nature of any enhanced support and could also lead to improvements in maintaining health and well-being. It will be interesting to see whether future studies comparing professional performance of SIMGs with locally trained doctors in specialty areas will corroborate some of the findings in this study. It should be noted that the colleague questionnaire used in this study was specifically adapted for use with surgeons from a previously validated tool accredited for general use in appraisal and revalidation by medical councils in other countries [20, 31]. The statistical analysis reported here provides initial evidence of the reliability and validity of the adapted questionnaire in surgical specialties, anda basis for future validation studies of colleague feedback on surgeons.

## Limitations of study

(a) Covid-19 interrupted data gathering processes, with normal clinician workflows affected by changes to provision of care during 2020. Covid-9 may also have impacted on perceptions and responses in unquantifiable ways. (b) The level of imputation required for certain items to raise the number of completed questionnaire responses, while statistically justified by missing value tests, will need to be reviewed before a subsequent study is repeated with the same items. (c) The study has focused on the specialist area of surgery in Australia and New Zealand. It is not known how applicable the results are to other jurisdictions and specialist areas. (d) Further analysis of comments from colleagues is required to identify possible qualitative differences in the perceptions of surgeons to support the quantitative differences reported here.

## Supplementary Information

**Additional file 1.**

**Additional file 2.**

## Data Availability

The datasets used during the current study are available from the corresponding author on reasonable request from an institutional address. Interested readers are also asked to contact the corresponding author for more details concerning the full content of the questionnaire and its layout.

## Abbreviations

IMG: International medical graduate
SIMG: Specialist international medical graduate
RACS: Royal Australasian College of Surgeons
CFET: Colleague Feedback Evaluation Tool
CFET-RACS: CFET tool modified for the RACSQ1: Demonstrating medical skills and expertise
Q2: Monitoring and evaluating care
Q3: Managing safety and risk
Q4: Considering options
Q5: Planning ahead
Q6: Implementing and reviewing decisions
Q7: Recognising where surgery may be needed
Q8: Maintaining dexterity and technical skills
Q9: Defining scope of practice
Q10: Having awareness and insight
Q11: Observing ethics and probity
Q12: Maintaining health and well-being
Q13: Caring with compassion and respect for patient
Q14: Meeting patient, carer and family needs
Q15: Responding to cultural and community needs
Q16: Gathering and understanding information
Q17: Discussing and communicating options
Q18: Communicating effectively
Q19: Documenting and exchanging information
Q20: Establishing a shared understanding
Q21: Playing an active role in clinical teams
Q22: Setting and maintaining standards
Q23: Leading that inspires others
Q24: Supporting others
Q25: Showing commitment to lifelong learning
Q26: Teaching supervision and assessment
Q27: Improving surgical practice
