# Supplementary material for: Are there differences between SIMG surgeons and locally trained surgeons in Australia and New Zealand, as rated by colleagues and themselves?
Source: BMC Med Educ. 2022 Jul 2;22:516. doi: 10.1186/s12909-022-03560-y (PMC9250230; doi:10.1186/s12909-022-03560-y)
Supplement: Supplementary file 1 — Additional file 1. [file 12909_2022_3560_MOESM1_ESM.docx]

## **Additional File 1: Statistical Methods**

Descriptive statistics used in this study include means, standard deviations, medians and quartiles. 95% upper and lower confidence intervals (CIs) are calculated through bootstrapping (taking multiple random samples from the dataset with replacement) using the 2.5^th^ and 97.5^th^ percentiles of the repeated bootstrap values.

Little’s test for missing completely at random, if significant, shows that missing values may be imputed from already existing values. Imputation was achieved in this study through regression and checked through reliability analysis so that all 2134 colleague responses could be used in those statistical analyses that required a full set of data.

Single measures intraclass coefficients (ICCs) are used to check for inter-rater reliability for this specific study. A two-way mixed effects model was chosen since each surgeon is rated by a different set of colleagues who were specifically selected by the surgeon from a larger population of possible colleagues and not drawn randomly.

Cronbach’s alpha measures (between 0 and 1) the internal consistency of a set of questionnaire items and estimates the reliability of the items as a scale. It is used as an indication as to whether the items measure the same construct through testing inter-relatedness of the items assuming that raters provide ratings on the same subject. Its use is reported in this study. But its application to studies involving multiple raters for a population of subjects (two-level analysis) is not clear, especially when there may be unbalanced (different number of raters per subject), fully nested (raters are unique to a subject) and uncrossed (raters provide only one rating) aspects of the sampling strategy to take into account. A signal-to-noise ratio measure specially designed for dealing with two-level psychometric data is also reported in this study, which takes into account variances among raters, items and subjects.

Analysis of variance (ANOVA) is used to test for differences in item ratings and averages in and between SIMG and Fellow data. Independent samples T-tests are used in this study to examine whether item means differ between the two surgeon groups. In addition, regression was used to control for the effects of demographic factors on colleague raw scores.

Principal component analysis (PCA) reduces the dimensionality of the data by creating uncorrelated variables (components) for maximizing the variance in the data. Varimax method is used for rotating and extracting the components, whereby each component has a small number of large loadings. The Kaiser-Meyer-Olkin (KMO) test is a sampling measure for indicating suitability for PCA. KMO values between 0.8 and 1.0 indicate that there are enough samples and sufficiently low variance for efficient identification of components. Bartlett test for sphericity indicates whether variables are sufficiently correlated for PCA, which provides a linear combination of variables into uncorrelated components that represent composites of the original variables. PCA is used in this study to explore the structure of the questionnaire items given that this is the first application of the questionnaire, and to identify how much variance in the total dataset at the aggregated (surgeon) level can be captured by the components.

Bivariate correlation checks for an association between two variables and whether it is possible to predict values of one variable given values of the other variable. If the two variables move in the same direction together, the correlation coefficient *r,* or ρ, is positive, otherwise it is negative. Correlations below 0.2 are considered weak and above 0.7 strong.

Psychometric network analysis explores relationships between variables, where nodes represent items and edges the associations between items. Partial correlations are used for edge calculation, where correlations between two variables are calculated after removing the effects of all other variables. Partial correlations are regularized to remove spurious connections through least absolute shrinkage and selection operator (LASSO) by minimizing the extended Bayesian information criterion (EBIC). Network analysis provides three centrality measures for determining importance of nodes. Degree centrality is the sum of absolute weights for a node and can indicate how node or symptom is directly activating or is activated by other nodes and symptoms. Closeness centrality indicates how quickly it takes a node to communicate with other nodes in the network depending on the strength of associations. A node with high closeness can be considered to interact quickly with other nodes. Betweenness centrality calculates how often a node is on the strongest route between two other nodes and may act as an intermediate or mediating node between other nodes. All centrality measures are calculated as standard deviations above and below mean 0 to allow comparisons to be made across different centrality measures. Identifying the most central nodes through summed centrality (sum of the standard deviations across all three centrality measures) may correspond to identifying the most central items for possible intervention and enhancement on the assumption that changes in central items should have greatest impact on other items. Such networks provide mechanisms for understanding performance at a system level using all items and for distinguishing groups through different item-interaction patterns. Differences in interaction patterns can lead to suggestions for improvement that are specific for a particular group.

References to sources are provided in the main text under Statistical Methods where appropriate.
