# Supplementary material for: Are there differences between SIMG surgeons and locally trained surgeons in Australia and New Zealand, as rated by colleagues and themselves?
Source: BMC Med Educ. 2022 Jul 2;22:516. doi: 10.1186/s12909-022-03560-y (PMC9250230; doi:10.1186/s12909-022-03560-y)
Supplement: Supplementary file 2 — Additional file 2. [file 12909_2022_3560_MOESM2_ESM.docx]

## **Additional File 2: Supplementary Tables and Figure**

Supplementary Table 1: Overview of colleague scores received by surgeons, item by item. The final column shows the difference in average item scores received (Fellows minus SIMGs).

Supplementary Table 2: Principal component analysis of aggregated (Surgeons) scores, with SIMGs (n=96) on the left and Fellows (n=25) on the right, showing one component for SIMG surgeons and three for Fellow surgeons. Maximum component loadings are shown in boldface.

Supplementary Table 3: Overview of self-assessment (SA) items for SIMGs and Fellows, with difference in the final column

Supplementary Figure 1: Comparison of self-scores against colleague scores by item and surgeon type. Note that the y-axis has been limited to the range 65 to 95 to make the differences clearer. Refer to Table 3 for a full description of the items.
